# Associations between levels of physical activity and satisfaction with life among Norwegian adolescents: a cross-sectional study

**DOI:** 10.3389/fspor.2024.1437747

**Published:** 2024-08-01

**Authors:** Erik Grasaas, Sergej Ostojic, Øyvind Sandbakk

**Affiliations:** ^1^ Teacher Education Unit, University of Agder, Kristiansand, Norway; ^2^ Department of Nutrition and Public Health, Faculty of Health and Sport Sciences, University of Agder, Kristiansand, Norway; ^3^ School of Sport Science, UiT The Artic University of Norway, Tromsø, Norway

**Keywords:** exercise habits, self-efficacy, well-being, school, mediation, public health

## Abstract

**Background:**

A robust association between physical activity (PA) and satisfaction with life (SWL) has been established, wherein self-efficacy has been identified as a mediator across different populations. However, there is a need to further examine the relationship between PA and SWL and whether self-efficacy act a as mediator within different levels of PA among Norwegian adolescents. Thus, the objective of this study was to explore the relationship between levels of PA and SWL by testing for self-efficacy as a possible mediator.

**Methods:**

Cross-sectional data from the 2022 Norwegian Ungdata Survey was utilized. Data included demographics and various health data that was collected anonymously. The electronic survey took place in classrooms and was administered by the respective teacher. Permission to access and use data was approved by the Norwegian Agency for Shared Services in Education and Research (SIKT). Statistical analyses were conducted using the PROCESS macro by Andrew Hayes for SPSS software.

**Results:**

Descriptive findings revealed that girls reported lower self-efficacy than boys (14.2 vs. 15.5, with a maximum of 20) and lower SWL (6.8 vs. 7.6, with a maximum of 10). About one out of five girls and one out of seven boys reported no days of weekly PA, whereas 4% of girls and 9.5% of boys adhered to the PA-recommendation of 60-min of daily exercise. Associations between PA levels and SWL was mediated by self-efficacy (all *p* < 0.05), with the highest indirect effect (56.3%) revealed in the association between those adhering to the PA-recommendations and SWL.

**Conclusions:**

Norwegian girls reported more sedentary behavior, less PA, lower self-efficacy, and lower SWL than boys across all grade levels. Mediation analysis revealed that up to 56.3% of the enhancement in SWL among those adhering to 60-min of PA recommendations was explained by increased self-efficacy. Norwegian government and policymakers should promote initiatives and regulations focusing on higher levels of PA to foster a resilient adolescent population with higher individual beliefs and higher subjective wellbeing.

## 1 Introduction

Physical activity (PA) is crucial for adolescent health (1). It is defined by the World Health Organization (WHO) as “any bodily movement produced by skeletal muscles that requires energy expenditure” (2). Research consistently shows that higher sedentary behavior increases the risk of various non-communicable diseases and reduces life expectancy (3–7). However, most adolescents do not meet the WHO recommendation of at least 60-min of moderate-to-vigorous intensity PA per day (2). Globally, about 8 out of 10 adolescents fail to meet PA recommendations, with girls being less active than boys (8). A recent Norwegian study found similar trends, with girls consistently reporting lower PA levels than boys (9). This study also highlighted strong links between PA levels and self-efficacy across genders and grade levels.

Self-efficacy, a concept developed by Bandura, plays a significant role in health behavior (10–13). It refers to one's belief in their ability to overcome obstacles (14). Previous research has explored various self-efficacy measures, which are all constrained to the specific task at hand (15). However, the general self-efficacy scale is a concept with a broader applicability, making it relevant to implement across genders and different dimensions of life. Self-efficacy and PA have a bidirectional relationship: PA increases self-belief and feelings of success, while self-efficacy influences activity choices and coping with barriers (16–18). Gender differences in PA levels among adolescents may be partly explained by variations in self-efficacy, with boys typically showing higher levels than girls (19–21). However, this relationship is complex (22), and self-efficacy is proposed as a mediator between efforts and health outcomes (23).

Satisfaction with life (SWL), a measure of subjective well-being, is positively influenced by PA in adolescents (24). SWL reflects one's overall life satisfaction, including all dimensions of life (25, 26). Studies have consistently found a positive relationship between PA and SWL, with sedentary behavior negatively impacting SWL (27, 28). Other relevant factors that are previously reported to affect PA and SWL in adolescence are socioeconomic status and stressor experiences (29, 30). Both PA and SWL tend to decrease during adolescence (31–34), highlighting the need to understand their underlying mechanisms. Higher self-efficacy is associated with higher SWL (31–33), suggesting a potential indirect pathway from PA to SWL through self-efficacy. In this specific context, self-efficacy stands out as a natural intermediate variable, due to the appliance to intrapersonal factors, such as the sense of achievement related to physical activity and interpersonal aspects related to observational learning and role modelling described by Bandura (14). Self-efficacy has been incorporated in several theoretical health behaviour models, such as in the health belief model and in the theory of planned behaviour (35, 36). Even though other societal factors and expectations, such as socioeconomic status and perceived stress, may also influence associations between PA and SWL (34, 37), this paper specifically focuses on self-efficacy to further our understanding of underlying behavioural mechanisms.

Previous models have suggested self-efficacy as a mediator between PA and SWL (38, 39), with emerging research exploring psychological mechanisms (40, 41). Recent studies among Chinese adolescents suggest that self-efficacy and stress management mediate the relationship between PA and mental health (42). According to Hayes, a mediator explains the process or mechanism through which the independent variable influences the dependent variable (43). Thereby, understanding how self-efficacy act between different levels of PA and SWL could inform interventions to enhance SWL by promoting PA, particularly among Norwegian adolescents.

This study aims to describe PA, self-efficacy, and SWL among Norwegian adolescents, to explore the relationship between PA and SWL with self-efficacy as a potential mediator. We hypothesize lower levels of PA, self-efficacy, and SWL in girls compared to boys, a positive association between PA and SWL, and a significant indirect role of self-efficacy in these associations.

## 2 Methods

### 2.1 Data collection

Ungdata is a national survey conducted yearly by Norwegian Social Research (NOVA) at Oslo Metropolitan University in collaboration with the regional center for drug rehabilitation (KoRus) and the municipal sector's organization (KS). Ungdata provides national reports every year, often comprised of data collected over the last three years. The annual survey entails a mandatory module; however the municipalities are provided with a set of optional questions they might consider incorporating in the survey (such as the self-efficacy measure). The municipalities provide information to their respective schools, and the survey is conducted electronically during a school hour. Non-participating adolescents are provided with other schoolwork task provided by their teacher. According to Ungdata, data retrieved from these surveys is well-suited for planning and initiating interventions related to adolescents and public health (44). Ungdata is financed from the Norwegian national budget through grants from the Norwegian Directorate of Health (44).

### 2.2 Study design and participants

This study employed cross-sectional data from the 2022 Norwegian Ungdata Survey, which included 108,843 participants. In 2022, all municipalities within the following Norwegian counties participated: Agder, Rogaland, Nordland, Akerhus and Østfold, as well as a selection of municipalities from the Møre and Romsdal county (45). In total, this included 124 Norwegian municipalities from Southern, Northern and mid-part of Norway, wherein most surveys were conducted in March and April 2022. The study includes Norwegian adolescents from lower (age 13–16 years) and upper secondary school (age 16–19 years).

### 2.3 Study variables

The Ungdata study comprises demographic measures and various health-related questions. Due to the survey's anonymity, age data is not available.

Levels of physical activity (PA) were assessed using the question, “Think about the last seven days. On how many days were you so physically active that you were short of breath or sweaty for at least 60 min in total in one day?”. Respondents could choose from five response alternatives ranging from “no days” (inactive), “1–2 days”, “3–4 days”, “5–6 days” and “7 days” (daily). Herein, we used “7 days” as a proxy for adhering with WHO recommendations of 60-min daily PA for adolescents (2). Single-item assessments of PA have exhibited robust reliability and validity (46), relevant in settings where extensive questionnaire or device-based measures are not feasible (47).

Self-efficacy was assessed using the Norwegian 5-item version of the General Perceived Self-Efficacy Scale (GSE) (48). GSE is a concept developed for evaluating the general confidence in one's abilities to cope with the upcoming challenges and demonstrated as a psychometric scale with high validity and reliability (49–51). Respondents are provided with five statements, rated on a scale from 1 (completely wrong) to 4 (completely right). A total score is summed ranging from 5 to 20, wherein higher scores indicate higher GSE levels.

Relevant covariates in regression models were socioeconomic status (SES), gender, perceived school stress as an indicator of psychological well-being, and over-the-counter analgesics (OTCA) use as an indicator of health status. Ungdata provides a validated construct for SES (52), which includes assessments of parental educational level and level of prosperity. Perceived school stress was assessed by the statement “*I get stressed by the schoolwork?”.* Responders were provided five response alternatives: “never”, “seldom”, “sometimes”, “often” and “very often”. OTCA use was assessed by using the question “*How often have you used non-prescription drugs (Paracet, Ibux and similar) during the last month?”*. Responders were provided five response alternatives: “never,” “less than once a week,” “at least weekly,” “several times a week,” and “daily”. Given the current study design and available variables from Ungdata, mediation analysis by Hayes was considered to be most fitting method for answering the research questions and to enlighten the underlying mechanisms.

### 2.4 Ethical consideration

The Norwegian Agency for Shared Services in Education and Research (ref. 821474), known as SIKT (53), has approved all questions used in Ungdata. Informed written consent was obtained from all responders and participation in the study was voluntary. Adolescents from the 1st grade (16 years or older) did not need parental consent, however adolescents from 8th to 10th grade need additional parental consent. All data are anonymous. The Ungdata survey is conducted in accordance with the Helsinki Declaration. This current study has structured the reporting according to the Strengthening the Reporting of Observational Studies in Epidemiology (STROBE) guidelines (54).

### 2.5 Statistical analyses

**Figure 1:**
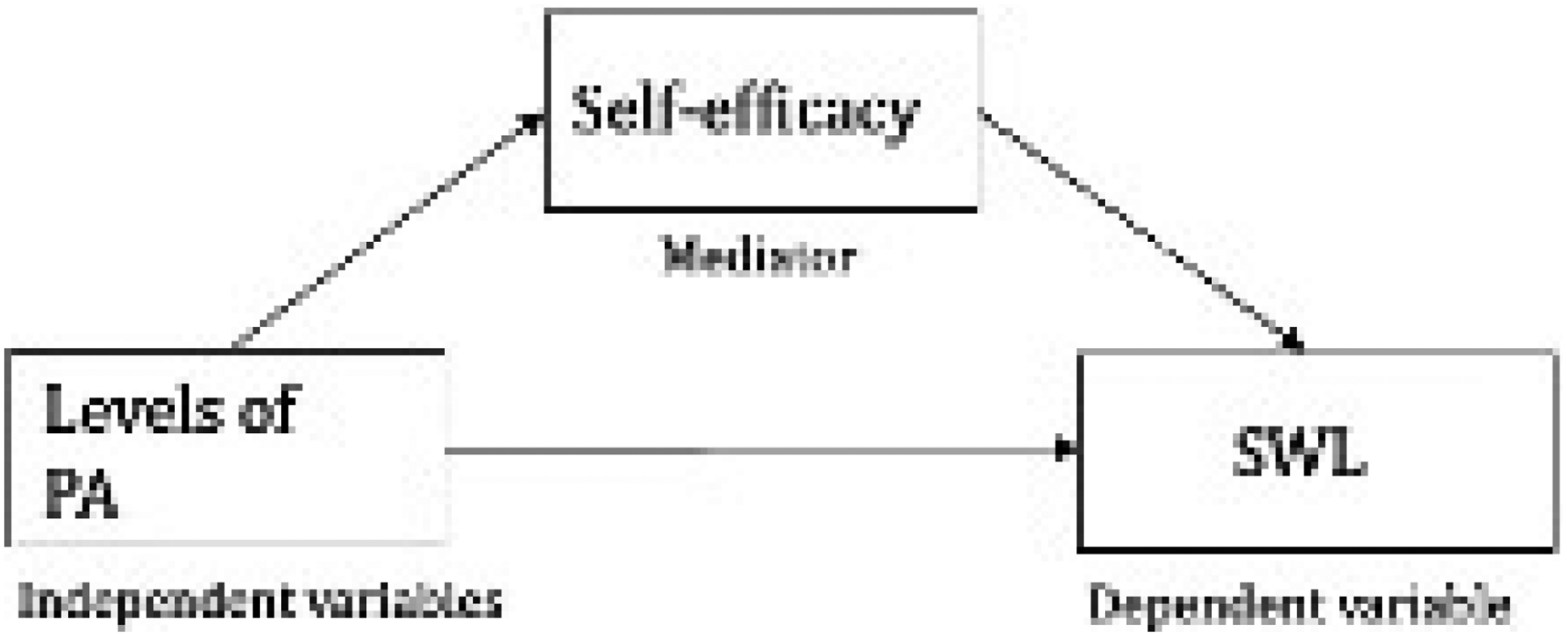
A simple meditation model of physical activity levels as independent variables, self-efficacy as mediator and satisfaction with life as dependent Variable.

All statistical analyses were performed using IBM SPSS Statistics for Windows, Version 25.0 (IBM Corp., Armonk, NY, USA). Descriptive measures for continuous variables are reported as means and corresponding standard deviation (SD). Categorical variables are presented as counts and percentages. Descriptive measures were stratified by gender and by grade levels (8th to 3rd grade). Multiple regression analyses were conducted to explore the association between levels of PA and SWL, and the association between levels of PA and self-efficacy. In addition, the association between self-efficacy and SWL was performed stratified by the levels of PA (Supplementary File S1). Simple dummy coding for the predicting categorical variables with binary indicators were performed. Visual inspection and kurtosis and skewness between −1 and 1 indicated a relative normal distribution of continuous study variables. Multiple regressions included covariates such as SES, gender, OTCA use and perceived school stress. Simple mediation analyses (Figure 1) were conducted using the PROCESS macro designed for SPSS by Andrew Hayes. Mediation analyses were entered with same covariates such as SES, gender, OTCA use and perceived school stress. The indirect effect was regarded statistically significant if the 95% confident interval (CI) did not include zero. According to Hayes, a calculation of the indirect effect does no longer need evidence of association between dependent and independent variable as a precondition for a mediation analysis (55), thus we included the non-significant association between PA “1–2 days” and SWL in the mediation analyses. However, opposite directions were revealed, making interpretation not feasible based on our assumptions of the causal path. The total effect (C) and direct effect (C') are presented in figures and the contribution of the indirect and direct effect is illustrated separately as percentage by dividing their effect on the total effect multiplied by 100. *P*-values <0.05 were considered statistically significant, and all tests were two-sided. Given the high response rate in the presented study variables and large sample size, imputation nor bootstrapping was not considered necessary.

## 3 Results

### 3.1 Participants

A total of 108,843 Norwegian school-based adolescents participated in Ungdata 2022, whereof 65,572 (60.2%) were responders from lower secondary school (8th to 10th grade) and 43,271 (39.8%) responders from upper secondary school (1st to 3rd year). The study sample comprised of 50.5% boys and 49,5% girls. Herein, 95,450 boys and girls reported their PA level, 18,868 their self-efficacy level and 103,653 their SWL, revealing a response rate of 87.7%, 97.5% and 95.2%, respectively.

### 3.2 Descriptive statistics

More girls than boys reported inactivity (no days of PA) (19.5% vs. 13.7%, respectively). The highest levels of inactivity were reported in the 2nd year for both genders (Tables 1 and 2). 13.6% of the girls and 21.2% of the boys reported being physical active 5–6 days a week. The highest relative difference in total scores between genders were revealed amongst the ones being physical active for minimum 60-min per day, herein 4.0% of the girls and 9.5% of the boys reported daily PA.

**Table 1:** Overview of study variables for girls stratified by grade level and total score.

Study variables	8th grade	9th grade	10th grade	1st year	2nd year	3rd year	Total score
Physical acitvity							
No days	17.3%	17.5%	18.2%	20.8%	23.3%	23.0%	19.5%
	*N* = 1,703	*N* = 1,645	*N* = 1,706	*N* = 1,731	*N* = 1,663	*N* = 940	*N* = 9,388
1–2 days a week	37.0%	34.9%	34.1%	37.1%	39.1%	39.4%	36.6%
	*N* = 3,643	*N* = 3,284	*N* = 3,208	*N* = 3,085	*N* = 2,787	*N* = 1,612	*N* = 17,619
3–4 days a week	28.7%	28.6%	27.3%	25.0%	23.2%	21.9%	26.4%
	*N* = 2,829	*N* = 2,692	*N* = 2,564	*N* = 2,075	*N* = 1,654	*N* = 897	*N* = 12,711
5–6 days a week	13.1%	14.9%	15.8%	13.2%	11.1%	12.1%	13.6%
	*N* = 1,286	*N* = 1,404	*N* = 1,485	*N* = 1,099	*N* = 794	*N* = 497	*N* = 6,565
Everyday	4.0%	4.2%	4.6%	3.8%	3.3%	3.6%	4.0%
	*N* = 390	*N* = 398	*N* = 435	*N* = 315	*N* = 232	*N* = 149	*N* = 1,919
Self-efficacy	13.9 (3.0)	13.8 (2.9)	14.1 (2.9)	14.4 (2.9)	14.6 (2.7)	14.6 (2.5)	14.2 (2.9)
Satisfaction with life	7.0 (1.9)	6.7 (1.9)	6.8 (1.9)	6.7 (1.9)	6.8 (1.8)	6.8 (1.7)	6.8 (1.9)

**Table 2:** Overview of study variables for boys stratified by grade level and total score.

Study variables	8th grade	9th grade	10th grade	1st year	2nd year	3rd year	Total score
Physical acitvity							
No days	14.1%	12.6%	12.6%	14.3%	15.2%	13.7%	13.7%
	*N* = 1,396	*N* = 1,181	*N* = 1,206	*N* = 1,187	*N* = 1,067	*N* = 420	*N* = 6,457
1–2 days a week	30.0%	26.8%	25.6%	29.0%	32.1%	30.3%	28.6%
	*N* = 2,978	*N* = 2,510	*N* = 2,453	*N* = 2,407	*N* = 2,257	*N* = 928	*N* = 13,533
3–4 days a week	29.4%	27.9%	26.2%	25.4%	25.9%	25.2%	27.0%
	*N* = 2,916	*N* = 2,611	*N* = 2,511	*N* = 2,105	*N* = 1,821	*N* = 773	*N* = 12,737
5–6 days a week	17.5%	22.2%	25.0%	22.0%	19.3%	21.1%	21.2%
	*N* = 1,737	*N* = 2,077	*N* = 2,395	*N* = 1,821	*N* = 1,355	*N* = 648	*N* = 10,033
Everyday	9.0%	10.5%	10.6%	9.3%	7.6%	9.7%	9.5%
	*N* = 888	*N* = 984	*N* = 1,013	*N* = 770	*N* = 535	*N* = 298	*N* = 4,488
Self-efficacy	15.2 (3.1)	15.4 (3.1)	15.6 (2.9)	15.6 (3.5)	15.8 (3.1)	15.9 (2.9)	15.5 (3.1)
Satisfaction with life	7.8 (1.7)	7.6 (1.7)	7.5 (1.8)	7.5 (1.8)	7.4 (1.7)	7.3 (1.7)	7.6 (1.8)

Lower mean (SD) total scores of self-efficacy were exhibited in girls [14.2 (2.9)] than in boys [15.5 (3.1)]. There was a tendency of increasing self-efficacy scores throughout the grade levels, with highest scores in 3rd year for both girls and boys [(14.6 (2.5) vs. 15.9 (2.9)]. Lower mean (SD) of SWL were reported in girls [6.8 (1.9)] than in boys [7.6 (1.8)], whereas the SWL scores remained consistent across the grade levels for girls and a small decrease among boys (Tables 1 and 2).

### 3.3 Regressions analyses

Regressions between the ones being inactive and SWL revealed an inverse association [B = −0.23; 95% CI (−0.31 to −0.15)] after adjusting for SES, gender, OTC analgesics use and perceived school stress (Table 3). Positive associations were revealed among adolescents reporting PA 3–4 times a week, 5–6 times a week or every day and SWL (B = 0.07, 0.08 and 0.08 respectively, all *P* < 0.01).

**Table 3:** Linear regressions of physical activity levels (independent) on satisfaction with life (dependent) adjusted for SES, gender, OTC analgesics use and perceived school stress.

Study variable	B	95% CI	*P* value
Physical activity levels			
** **No days	−0.23	−0.31 to −0.15	<0.01
** **1–2 days a week	0.03	−0.01 to 0.07	0.13
** **3–4 days a week	0.07	0.04 to 0.09	<0.01
** **5–6 days a week	0.08	0.06 to 0.10	<0.01
** **Active everyday	0.08	0.06 to 0.09	<0.01

Regressions between the ones with no days of PA and self-efficacy revealed an inverse association [B = −0.76; 95% CI (−1.21 to −0.30)] that remained significant after adjusting for SES, gender, OTC analgesics use and perceived school stress (Table 4). Significant positive associations with self-efficacy were revealed amongst adolescents reporting PA 5–6 days a week and PA every day (B = 0.16 and 0.14, respectively). In addition, self-efficacy was positively associated with SWL across all PA levels (all *p* < 0.01) after adjusting for SES, gender, OTC analgesics use and perceived school stress (Supplementary File S1).

**Table 4:** Linear regressions of physical activity levels (independent) and self-efficacy (mediator) adjusted for SES, gender, OTC analgesics use and perceived school stress.

Study variable	B	95% CI	*P* value
Physical activity levels			
** **No days	−0.76	−1.21 to −0.30	<0.01
** **1–2 days	−0.09	−0.31 to 0.14	0.46
** **3–4 days	0.09	−0.06 to 0.24	0.25
** **5–6 days	0.16	0.04 to 0.27	<0.01
** **Active everyday	0.14	0.05 to 0.24	<0.01

### 3.4 Mediation analyses

**Figure 2:**
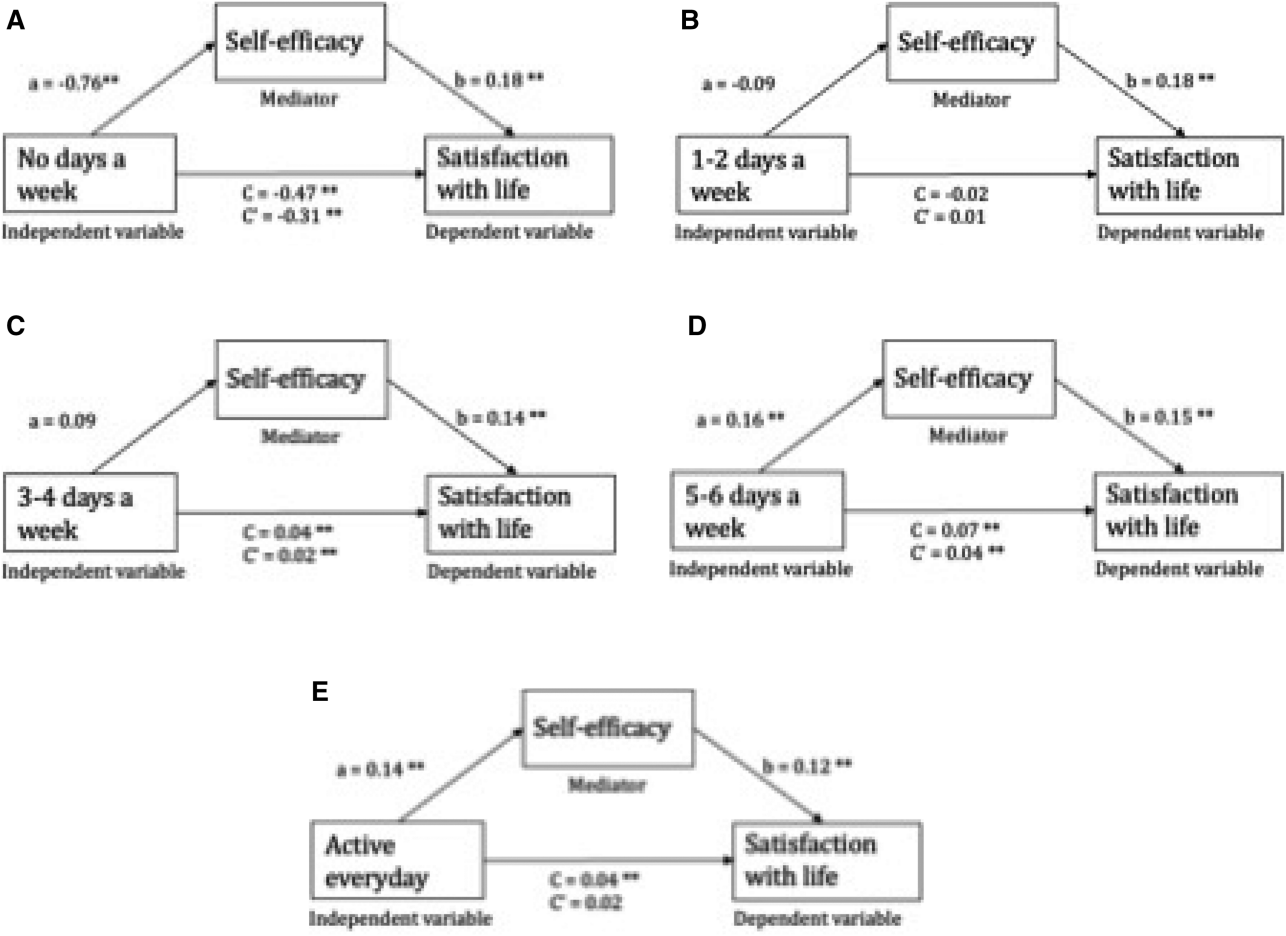
Mediation by self-efficacy of the association between different physical activity levels and satisfaction with life. Path a and b illustrates the mediating effect through self-efficacy, expressed as indirect effect. The direct effect (C’) represents the isolated effect of the independent variable on the dependent variable. The total effect (**C**) represents the combined effect of the direct and indirect effects. **p < 0.01, *p < 0.05.

Self-efficacy had a significant indirect effect across all models (*p* < 0.05), after adjusting for SES, gender, OTC analgesics use and perceived school stress. The highest indirect effect in the association between PA and SWL was identified amongst the adolescents being physical active everyday (Table 5), herein 56.3% of the increase in SWL could be explained by the indirect effect (improvement in self-efficacy). This association was mediated by self-efficacy by revealing a non-significant direct effect (Figure 2).

**Table 5:** Direct and indirect effects presented as percentage.

Physical activity levels	Direct effect %	Indirect effect %
** **No days	66.8	33.2
** **1–2 days	n.a	n.a
** **3–4 days	59.7	40.3
** **5–6 days	57.2	42.8
** **Active everyday	43.7	56.3

A tendency of higher indirect effect amongst adolescents being more frequently physical active was found, revealing an indirect effect of 40.3% (95% CI 0.010 to 0.021), 42.8% (95% CI 0.026 to 0.035), and 56.3% (95% CI 0.016 to 0.032) amongst adolescents being physical active 3–4 for days a week, 5–6 days a week and physical active every day, respectively. The reduction in SWL amongst the inactive adolescents was explained by an indirect effect of 33.2% (95% CI −0.177 to −0.133), whereas the majority of the association was explained by the direct path (66.8%) between inactivity and SWL. The opposite directions of indirect and direct effect amongst adolescents being physical active 1–2 days a week, made interpretation not feasible based on our assumptions and was therefore not included in Table 5.

## 4 Discussion

In this study, our aim was to describe PA, self-efficacy, and SWL among Norwegian adolescents, and to explore the relationship between PA and SWL with self-efficacy as a potential mediator. Our descriptive findings underscored that girls reported lower levels of PA, self-efficacy, and SWL than boys. Regression analyses revealed that the associations between PA levels and SWL were mediated by self-efficacy, with the highest indirect effect mediating the association between those adhering to PA recommendations and SWL. These findings suggest that higher life satisfaction among physically active adolescents can be explained by an increase in self-efficacy, while lower life satisfaction among inactive adolescents can be explained by a decrease in self-efficacy.

Our findings revealed that most Norwegian adolescents failed to meet PA recommendations, which aligns with previous global findings (8, 56). However, a notable difference in prevalence across countries and cultures has been reported, although lower PA levels among girls compared to boys seems to be consistent (56). Previous findings across Europe have reported about two-thirds of European adolescents being inadequately physically active (57). In Norway, Steene-Johannessen and colleagues revealed that about half of 15-year-old adolescents adhered to PA recommendation, assessed with objective accelerometer measures (58). Furthermore, a recently published study based on Norwegian Ungdata material from 2017 to 2021 revealed PA adherence ranging from 15% to 30% in adolescents, with girls consistently exhibiting lower adherence to PA recommendations compared to boys (9). As hypothesized, our study also unveiled that girls report lower PA levels than boys, with the highest relative differences in the most active category of minimum 60-min of daily physical activity.

However, it is important to highlight methodological considerations when interpreting these results, as studies may operate with operational definitions of adherence to PA recommendations based on different measurements, age variations, and sample sizes. Thus, comparisons should be interpreted with caution. For instance, previous Ungdata surveys did not include a category of a minimum of 60-min of daily physical activity. Therefore, the previous study from 2017 to 2021 examining the adherence of PA among this population used the category “at least five times a week” as a proxy for PA recommendations (9). Thereby, our findings among girls and boys seem to be aligned with previous years’ proportions of Norwegian adolescents being active at least 5–6 days or more often. However, by utilizing the new single-item category of adhering to a minimum of 60-min of daily PA in this current study, findings revealed that only 4% of girls and 9.5% adhered to PA recommendations. We argue that the category of a minimum of 60-min of daily PA aligns closer to WHO recommendations for PA and thus presumably indicates an underestimation of previous findings, which most prominently conveys a worrying status among the proportion of Norwegian adolescents failing to adhere to the PA recommendations.

Our findings of lower life satisfaction and self-efficacy in girls are in accordance with our hypothesis and with previous evidence among Norwegian adolescents (21, 29, 59, 60). There are several factors potentially influencing the gender disparities, such as differences in peer relations, academic aspirations and/or pressure of appearance and performance. A recent study by Kleppang and colleagues examined the variance in self-efficacy among Norwegian adolescents (61), in which the items “felt mastering things” and “felt useful” revealed the strongest contributions to the variance in self-efficacy, followed by “support from friends” and “parental support”. Societal expectations, both by others and themselves, might impact girl's self-efficacy and resilience. Earlier findings have suggested that higher self-efficacy in adolescence underscores higher resilience (62), which is can be crucial for coping with everyday stressors and thereby impacting adolescents' perception of life satisfaction (29).

As hypothesized, a positive association between higher PA levels and SWL was revealed, and a significant indirect role of self-efficacy were identified in these associations. The robust findings of a positive impact of higher PA on SWL and the inverse association between inactivity and SWL aligns with previous studies (27, 28). Furthermore, Deng and colleagues investigated the relationship between PA and SWL among university students in China and explored the mediating role of self-efficacy and resilience, and found that both self-efficacy and resilience mediated this relationship (41). Thereby, PA does not only enhance SWL directly, but also indirectly by improving their self-efficacy and resilience (41). Similar findings has been revealed in adolescents, revealing both self-efficacy and stress-management as chain mediation effect on the relationship between PA and mental health (42). The authors recommend that future research should aim to identifying types of physical activities that has the greatest potential to enhance self-efficacy (42). Examining how various types of physical activities, considering factors such as duration, intensity, and frequency, may contribute to enhancing self-efficacy among adolescents is intriguing, as it could offer more targeted recommendations and guidelines for physical activity in a school-based setting. However, despite the potential insights that such research could provide, our study's robust findings highlight the significant impact of higher levels of physical activity on life satisfaction, particularly through the mechanism of increased self-efficacy. This underscores the importance of implementing more physical activity programs in Norwegian lower and upper secondary schools to nurture a resilient upcoming population. From a psychological perspective, our findings further strengthen the theoretical view of self-efficacy acting as an explanatory health behavior variable that increases during high levels of physical activity and boost the feeling of success and thereby life satisfaction. Accordingly, adolescents with low levels of physical activity seems to have low resilience and belief in their own capacity, and thereby lower life satisfaction.

### 4.1 Strengths and limitations

Several strengths have contributed to the robustness of this study. However, foremost among the limitations is the use of a single-item measure for assessing PA, which should be acknowledged. Employing more comprehensive measures, such as validated questionnaires or objective measures, could have enhanced the validity of the data. Nonetheless, it is worth noting that the category of a minimum of 60-min of daily exercise aligns well with the WHO's guidelines for PA among adolescents, which can be considered a strength, particularly as our findings suggested that those adhering to these guidelines exhibited the highest indirect effect of self-efficacy. Furthermore, the study benefits from a large sample size and a low number of missing data, ensuring a high level of representativeness and supporting the validity of the study (52). Another strength is the inclusion of a validated variable for socioeconomic status provided by Ungdata, a well-known Norwegian survey with established procedures for data collection and cleaning (52). In addition, ninety-seven percent of the adolescents participating in the 2022 Ungdata survey reported that they answered honestly to the questions and eighty-seven percent reported that the questions were easy to answer (45). Additionally, adherence to STROBE guidelines enhances transparency and accurate reporting in the study.

The optional nature of the self-efficacy measure for municipalities resulted in fewer adolescents being able to report, as only 20 out of 124 municipalities included this measure, leading to a lower sample size, which should be considered a limitation. Additionally, our mediation model is constructed based on theory, research evidence, and our own assumptions. Therefore, there is no guarantee that the directional nature of the model is accurate, as there may be opposing directions. While we explored self-efficacy as a potential causal pathway between PA levels and life satisfaction, we do not claim to establish causality. Furthermore, the cross-sectional design of the study does not allow for understanding over time, and there may be a risk of recall bias as adolescents are asked to assess their PA levels over the last seven days. Moreover, ideally other relevant co-variates, such as BMI, comorbidities, and other health-related variables could have been included.

### 4.2 Clinical implications

This study contributes to the field by elucidating the underlying mechanisms of self-efficacy in the relationship between PA levels and life satisfaction among Norwegian adolescents, while adjusting for several covariates. From a public health perspective, these findings imply that the Norwegian government should prioritize initiatives and regulations aimed at promoting increased physical activity among school-based adolescents. This could involve incorporating more mandatory physical education in both lower and upper secondary schools, along with integrating more daily activity across various school subjects to foster a general higher activity level. Moreover, our findings highlight the clear negative consequences of inactivity, leading to lower life satisfaction attributed to a decrease in self-efficacy. Considering the potential benefits of cultivating a resilient adolescent population through increased mandatory physical activity in Norwegian schools, we strongly advocate for policy and practice efforts to be combined, thereby promoting higher individual beliefs and greater subjective well-being among Norwegian adolescents.

## 5 Conclusions

In this current study, Norwegian girls consistently reported more sedentary behavior, less PA, lower self-efficacy, and lower SWL compared to boys across all grade levels. Regression analyses indicated a positive association between PA and SWL, with self-efficacy playing a significant indirect role in these associations. Mediation analysis further demonstrated that up to 56.3% of the improvement in SWL among those adhering to the 60-min PA recommendations was attributed to increased self-efficacy. The implications of this study are pertinent for the Norwegian government and policymakers, emphasizing the need to promote initiatives and regulations aimed at increasing PA to cultivate a resilient adolescent population with higher individual beliefs and greater subjective well-being.

## Data Availability

Publicly available datasets were analyzed in this study. This data can be found here: Data supporting the results of this study is available upon request from the Norwegian Agency for Shared Services in Education and Research (SIKT) (45). Reference to dataset from SIKT: NOVA & Bakken, Anders. (2024). Ungdata 2010-2023 https://doi.org/10.18712/NSD-NSD3157-V1.
