# Supplementary material for: Associations between levels of physical activity and satisfaction with life among Norwegian adolescents: a cross-sectional study
Source: Front Sports Act Living. 2024 Aug 1;6:1437747. doi: 10.3389/fspor.2024.1437747 (PMC11324472; doi:10.3389/fspor.2024.1437747)
Supplement: Supplementary file 1 [file Table1.docx]

**Supplementary File 1**

Linear regressions of self-efficacy (mediator) and satisfaction with life (dependent) stratified by physical activity categories adjusted for SES, gender, OTC analgesics use and perceived school stress.

| Study variable | B | 95% CI | P value |
| --- | --- | --- | --- |
| Self-efficacy |  |  |  |
| No days  1-2 days  3-4 days  5-6 days  Active everyday | 0.18  0.18  0.14  0.15  0.12 | 0.16 to 0.20  0.16 to 0.19  0.15 to 0.18  0.13 to 0.17  0.09 to 0.15 | <0.01  <0.01  <0.01  <0.01  <0.01 |
